# Supplementary material for: Causes and consequences of sex-chromosome turnovers in Diptera
Source: Evol Lett. 2026 Mar 12;10(3):253–68. doi: 10.1093/evlett/qrag003 (PMC13231274; doi:10.1093/evlett/qrag003)
Supplement: qrag003_Supplemental_Files [file qrag003_supplemental_files.zip › Supplementary_Datasets.zip!/Supplementary_Datasets/Dataset_2_Graphical_representation_outgroups/Drosophilidae_Drosophila_elegans_chromosomes.pdf]

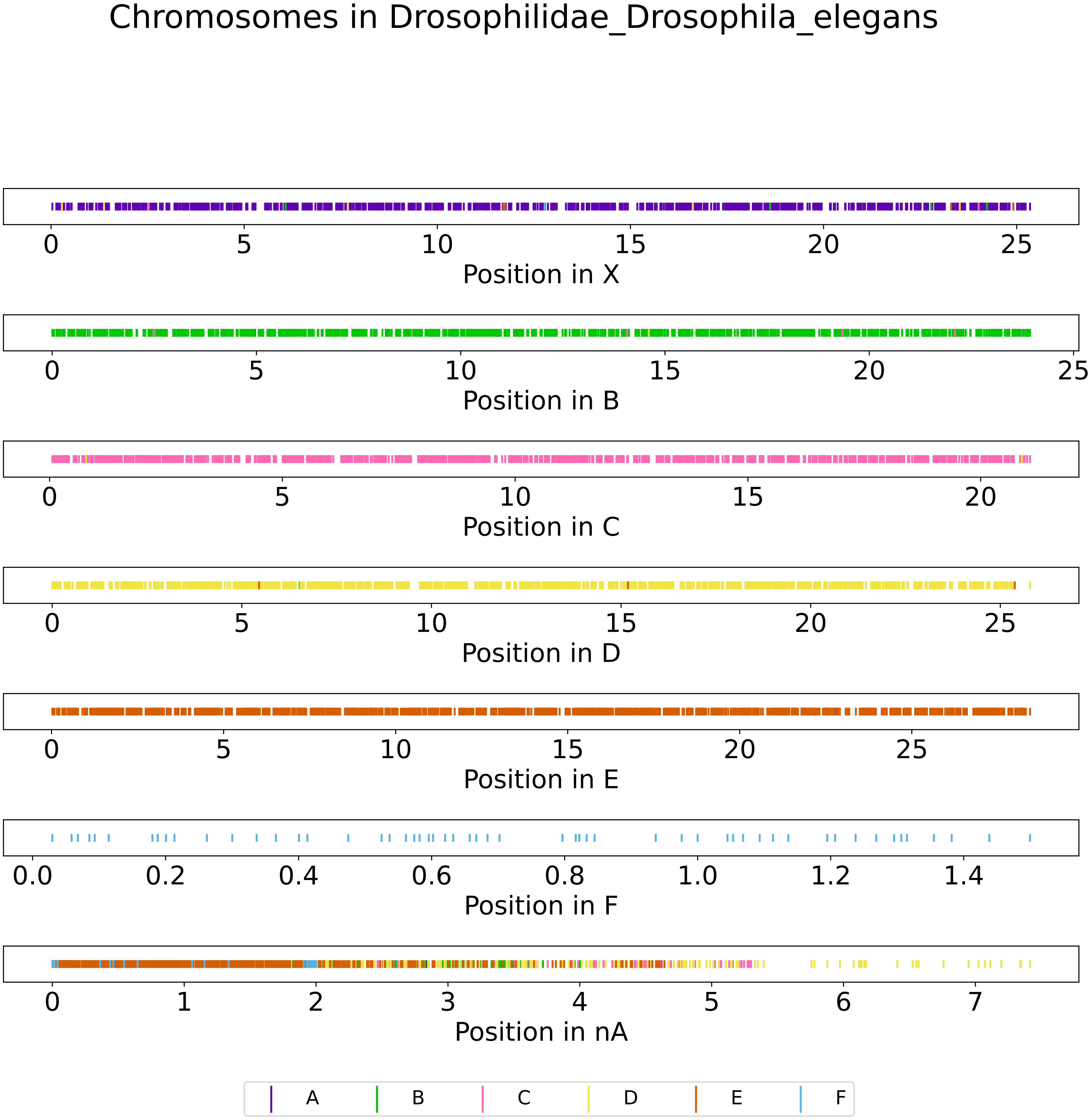
Chromosomes in Drosophilidae_Drosophila_elegans
0 5 10 15 20 25
Position in X
0 5 10 15 20 25
Position in B
0 5 10 15 20
Position in C
0 5 10 15 20 25
Position in D
0 5 10 15 20 25
Position in E
0.0 0.2 0.4 0.6 0.8 1.0 1.2 1.4
Position in F
0 1 2 3 4 5 6 7
Position in nA
A
B
C
D
E
F
